# Guillain-Barre Syndrome and Syndrome of Inappropriate Antidiuretic Hormone (SIADH) Secretion as Paraneoplastic Syndromes in Splenic Marginal B-cell Non-Hodgkins Lymphoma: A Rare Presentation

**DOI:** 10.7759/cureus.10133

**Published:** 2020-08-30

**Authors:** Madhuri Patil, Vijayadershan Muppidi, Sreenath Meegada, Keanan T Dowell, Joe D Bowers

**Affiliations:** 1 Internal Medicine, CHRISTUS Good Shepherd Medical Center, Longview, USA; 2 Internal Medicine, Indiana University Health, Indianapolis, USA; 3 Internal Medicine, University of Texas Health Science Center/Christus Good Shepherd Medical Center, Longview, USA; 4 Neurology, CHRISTUS Good Shepherd Medical Center, Longview, USA

**Keywords:** splenic marginal zone lymphoma, guillain barre’s syndrome (gbs), siadh, non hodgkin's lymphoma, paraneoplastic syndromes

## Abstract

Splenic marginal zone lymphoma (SMZL), a rare sub-type of non-Hodgkin lymphoma (NHL) presents with abdominal discomfort, lymphocytosis, cytopenias along with B symptoms including fatigue, night sweats, night fevers, weight loss. NHLs rarely present with paraneoplastic neurological syndromes like Guillain-Barre (GB) syndrome, myelopathy causing paraplegia, chorea, neuromyotonia, vasculitic neuropathy and dermatomyositis. Here, we present a 85-year old caucasian lady presenting with GB syndrome and Syndrome of Inappropriate Antidiuretic Hormone (SIADH) who eventually got diagnosed with SMZL.

## Introduction

Splenic marginal zone lymphoma (SMZL) is a subtype of Non-Hodgkin's lymphoma (NHL). Marginal zone lymphomas arise from B-lymphocytes and the name is derived due to its origin from the marginal zone of secondary lymphoid follicles [1]. Marginal zone lymphomas can be nodal, extranodal or mucosa-associated lymphatic tissue and splenic in origin. Median age of SMZL incidence is 65-70 years; it is not common before 50 years of age. It is more common in Caucasians compared to other races, with no gender disparity. It can be associated with other autoimmune disorders, asthma, and chronic hepatitis C Virus infection [1]. 

## Case presentation

An 85-year old Caucasian female presented to our facility from home with three days of nausea, vomiting, and abdominal pain. Past medical history includes hypertension, diverticulosis and osteoarthritis. Symptoms came on spontaneously, with no aggravating or alleviating factors. She vomited twice prior to presentation, with nonbloody and nonbilious vomitus. She described her abdominal pain as generalized, dull and aching in nature, and constant over these three days. She experienced one week of constipation, and denied any diarrhea, melena, or hematochezia. Additionally, she endorsed generalized weakness, body aches (most notably in her legs), and intermittent headache that had been persisting for about four weeks.

**Figure 1:**
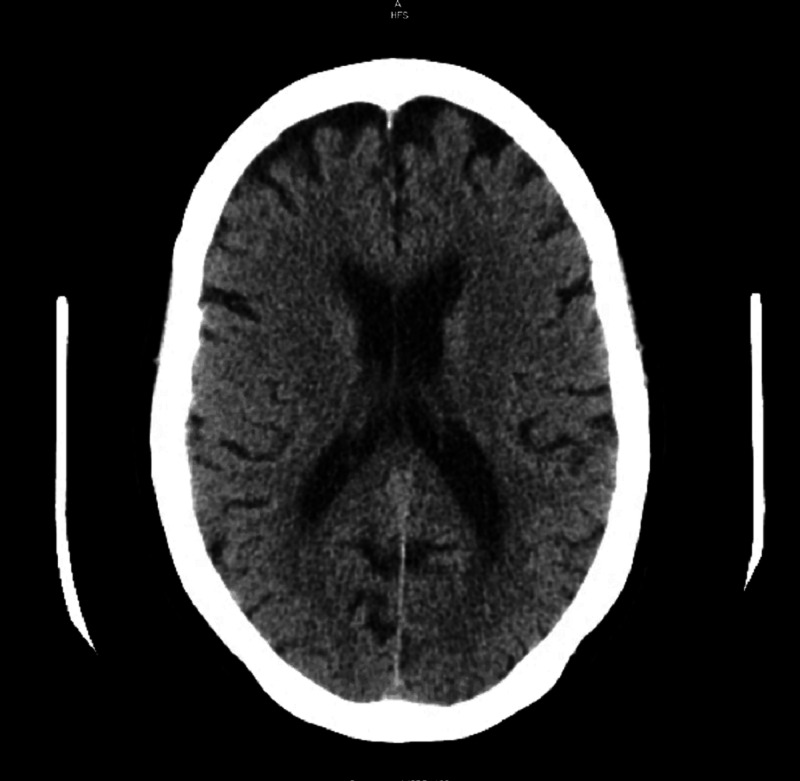
Computed tomography Scan of Head with out contrast showed no acute abnormalities.

On presentation, her physical exam consisted of a blood pressure of 173/78 mmHg but otherwise normal vital signs, normal neurologic examination, and tenderness to palpation in the left lower quadrant. Initial laboratory workup revealed an elevated white blood cell count of 12,300/ul of blood with 51% lymphocytes on differential, low sodium at 127 milliequivalents per liter, potassium at 3.5 milliequivalents per liter, chloride at 95 milliequivalents per liter, creatinine at 0.42 milligram/deciliter, AST at 88 U/L, ALT 99 U/L, alkaline phosphatase at 119 U/L, total bilirubin at 0.8 mg/dL, calcium at 7.8 mg/dL, and albumin at 3.2 g/dL. Lipase was normal at 60 U/L, serum osmolality was 250 milliosmoles/kilogram of water, urine osmolality was 280 milliosmoles/kilogram of water, thyroid stimulating hormone and serum cortisol at 5 AM was normal at 2.0 milliunits/liter and 16 ug/dL respectively. Computed tomography (CT) of the head revealed no acute abnormalities (Figure 1)

**Figure 2:**
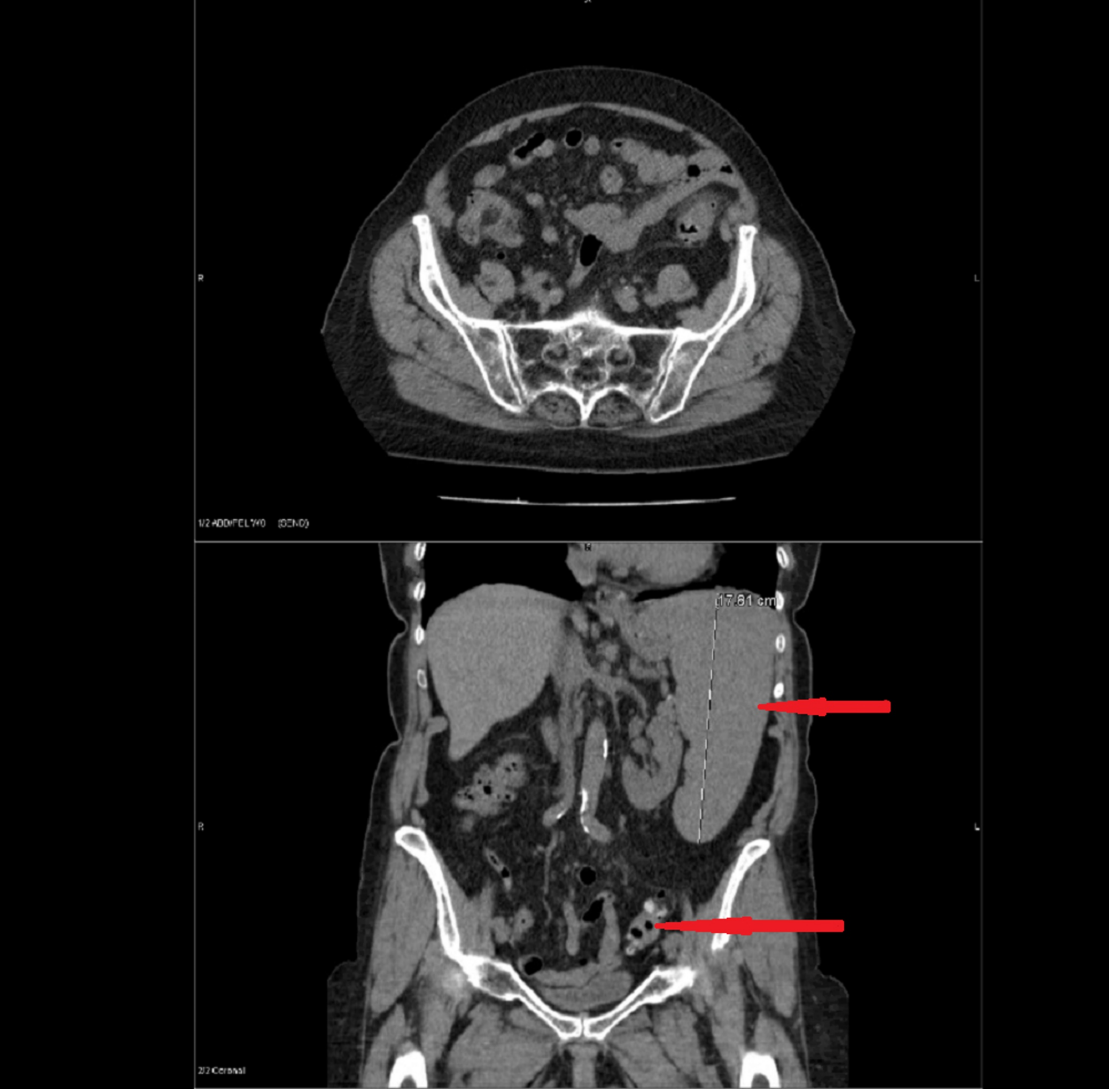
Computed tomography scan abdomen and pelvis with intravenous contrast showed Splenomegaly (Red arrow 1) and Sigmoid diverticulitis (Red arrow 2)

CT of the abdomen and pelvis revealed an enlarged spleen measuring 17.8 cm as well as diverticulitis of the descending and sigmoid colon with colonic wall thickening and surrounding inflammation (Figure 2).

**Figure 3:**
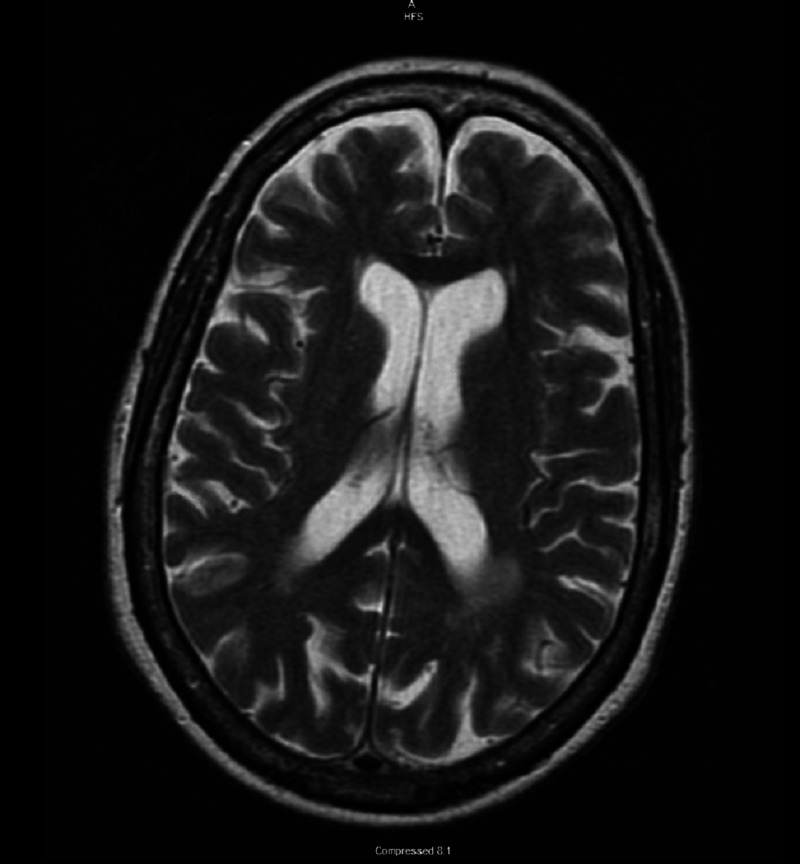
Magnetic resonance imaging (T2 hyperintensity) of brain showed non-specific white matter changes, no acute abnormalities.

Intravenous metronidazole and levofloxacin were started for suspected diverticulitis. The day after admission, she developed left arm weakness and magnetic resonance imaging (MRI) of the brain was ordered. The findings of the MRI were nonspecific, described as mild T2 hyperintensity in the periventricular white matter with non-specific changes (Figure 3).

After two days, the patient began to experience lower extremity weakness and was unable to walk without assistance. The following day, she had right facial droop and was alert with dysarthria and incomplete right lid closure. On exam, she had diffuse motor weakness of bilateral upper and lower extremities, intact sensation, and absent deep tendon reflexes. Neurology was consulted and lumbar tap was ordered. Cerebrospinal fluid (CSF) was clear and colorless and analysis revealed albumin 100 mg/dL, albumin (MS) 2950 mg/dL, IgG 22 mg/dL, no oligoclonal bands, white blood cells 1/mm^3^, and red blood cells 1/mm^3^, with an opening pressure of 6 cm H_2_O.

Additionally during hospital stay, the cause of the patient’s splenomegaly and lymphocytic leukocytosis were evaluated by examining peripheral blood. Flow cytometric immunophenotypic analysis of the same showed 48% small kappa clonal B-cell population expressing Cluster of differentiation (CD) 19 and moderate CD20; the sample was negative for CD5, CD10, CD25 and CD103. About 27.5% neutrophils with <0.1 % myeloblasts were identified. These findings are consistent with B-cell, non-Hodgkin lymphoma cells. The patient received intravenous immune globulin (IVIG) for five days as well as supportive care with physical and occupational therapy for treatment of Guillain-Barré Syndrome. Her strength improved and she was ultimately discharged to inpatient rehab with subsequent oncology follow up.

After discharge, she experienced B symptoms including fatigue, fever, and sweats. She was later seen outpatient by oncology, bone marrow biopsy was performed, and she was ultimately diagnosed splenic marginal zone lymphoma. She was started on rituximab. On follow up evaluation several months later and after several infusions of rituximab, she was noted to have complete resolution of these B symptoms and splenomegaly.

## Discussion

About one-fourth of the patients with SMZL are asymptomatic [2]. In the remaining patients, symptoms depend on site of involvement and lab abnormalities. Abdominal pain or discomfort is the most common symptom and is due to splenomegaly [3]. Lymphadenopathy is rare, except splenic hilar lymph nodes and extranodal involvement is usually limited to liver or bone marrow [2]. Although B symptoms and hyperviscosity syndromes are not common, presence of B symptoms especially with an elevated lactate dehydrogenase (LDH) should raise suspicion for transformation to diffuse large B cell lymphoma seen in about 10% of cases [3]. Angioedema is a rare feature if patients develop C1 esterase inhibitor deficiency [2].

Lymphocytosis or cytopenias are usually found in the laboratory findings. Isolated lymphocytosis is seen in asymptomatic patients [3]. Cytopenias are usually due to hypersplenism and sometimes due to bone marrow infiltration. Most common cytopenia is anemia, which is more commonly due to increased destruction from hypersplenism, but can also occur secondary to hemolysis from autoantibodies and less frequently due to decreased production from bone marrow infiltration [3]. Other lab abnormalities include elevation of LDH and monoclonal protein such as immunoglobulin M kappa [2]. In about 20% of the patients, a variety of associated autoimmune disorders are reported which include autoimmune hemolytic anemia, cold agglutinin disease, thrombocytopenia, anticoagulants, primary biliary cirrhosis and rheumatoid arthritis [2, 3].

Several paraneoplastic neurological syndromes (PNSs) have been reported in both Hodgkin’s lymphoma and NHL [4]. There are a few case reports in the literature regarding paraneoplastic disorders in splenic marginal zone lymphomas such as paraneoplastic paraplegia [5]. Guillain-Barre syndrome is rarely associated with NHL as drug toxicity and CNS infiltration are more common causes of neuropathology [6]. Molecular mimicry between nervous tissue and tumor angtigens with subsequent immune complex mediated damage causes PNSs [7]. Our patient had GBS associated with a splenic marginal zone lymphoma with symptoms of weakness of extremities and facial droop. Based on the clinical features, CSF findings and the response to IV Immunoglobulin, GBS was confirmed in our case.

Another rare feature in our case is syndrome of inappropriate antidiuretic hormone secretion (SIADH) seen in splenic marginal zone lymphoma. She had hyponatremia at presentation. Urine studies were very suspicious for SIADH and sodium levels improved with free water restriction. Both these features suggest SIADH as the etiology of hyponatremia in our case. Although there are a few cases of SIADH reported in NHL, the exact mechanism of SIADH is not clear in our case [7]. It is not clear if the SIADH is secondary to potential triggers such as nausea and pain, or due to lymphoma itself.

Other uncommon PNSs seen in NHLs are paraneoplastic chorea, opsoclonus-myoclonus, paraneoplastic myelopathy, sensory neuronopathy, autonomic ganglionopathy, sensorimotor neuropathy, vasculitic neuropathy, neuromyotonia, Lambert-Eaton myasthenic syndrome, myasthenia, and dermatomyositis [8-19].

## Conclusions

Paraneoplastic syndromes are uncommon in Hodgkins lymphoma and NHLs. High index of suspicion and thorough neurological evaluation should be done judiciously to confirm the diagnosis. To the best of our knowledge, GB syndrome and SIADH in combination has never been discussed in the literature till date.
